# A systematic review of the implementation of healthy food retail interventions in settings with multiple food retail outlets (complex food retail settings)

**DOI:** 10.1017/jns.2024.52

**Published:** 2024-09-18

**Authors:** Adyya Gupta, Catherine E. Huggins, Gary Sacks, Joel Gittelsohn, Anna Peeters

**Affiliations:** 1 Deakin University, Institute for Health Transformation, Global Centre for Preventive Health and Nutrition, School of Health and Social Development, Faculty of Health, Geelong, VIC 3220, Australia; 2 Department of International Health, Centre for Human Nutrition, Johns Hopkins Bloomberg School of Public Health, Baltimore, MD, USA

**Keywords:** Complex food retail settings, Food purchase, Food retail, Health promotion, Implementation science

## Abstract

Complex food retail settings, where multiple food retail outlets operate in close proximity are common. Despite their ubiquity, there remains a significant knowledge gap regarding healthy food retail interventions implemented within these settings. Furthermore, understanding the factors affecting the implementation of interventions in these settings remains limited. This systematic review aimed to (1) identify and describe complex food retail settings where interventions were implemented to promote the healthiness of foods purchased, (2) synthesise the evidence on the effectiveness of the interventions implemented, and (3) identify enablers and barriers to the implementation of the interventions in these settings. Four databases, namely, MEDLINE Complete, Global Health, Embase, and Business Source Complete, were searched until December 2022. The Effective Public Health Practice Project quality assessment tool was used. Six studies reported on the implementation of interventions promoting healthy food purchases across multiple food retail outlets. Three studies each described two complex food retail settings: university and hospital. Interventions including promotion and promotion plus price improved the healthiness of foods purchased. There was limited description of institutional food policies, conceptual frameworks, formative research, or evaluation outcomes to inform the implementation of interventions in these settings. No study analysed enablers and barriers to the implementation of interventions. No study identified their settings as complex food retail settings. There is limited evidence describing complex food retail settings, their impact on intervention effectiveness, and associated enablers or barriers. Investigating factors influencing the effectiveness of interventions implemented within complex food retail settings is critical to support their implementation at scale.

## Graphical abstract

**Figure:**
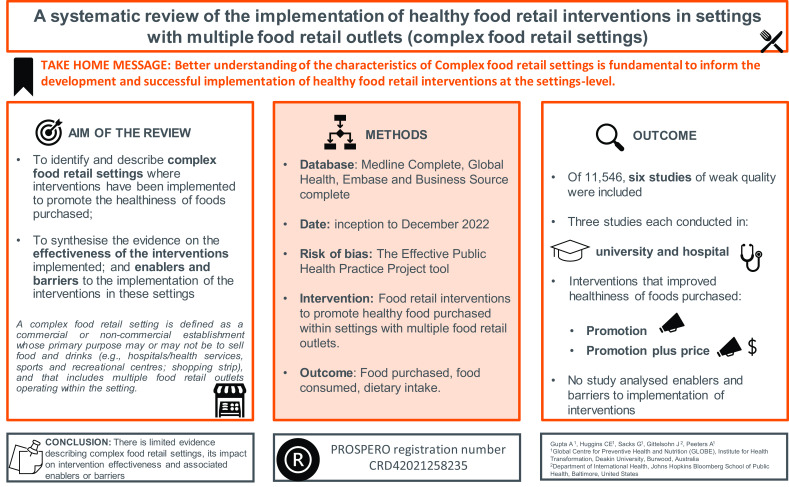

## Background

Unhealthy diet is a leading risk factor for non-communicable diseases that causes substantial societal and economic burden.^(1)^ Food environments are known to drive dietary choices and associated population health outcomes.^(2)^ There is a comprehensive body of evidence that has tested the effectiveness of marketing mix strategies^(3)^ implemented in single food retail outlets such as a supermarket or cafe on diet-related outcomes^(4)^ including consumer purchasing behaviour, dietary intake, and health. For example, front-of-package labels and point-of-sale signage are among the most commonly and successfully implemented strategies in single food retail outlets worldwide to inform healthy food choices.^(5)^ There is also evidence illustrating a range of factors at different levels of the socio-ecological model that influence the implementation, sustainability, and scalability of these strategies in single food retail outlets,^(6)^ both from consumer and retailer perspectives^(7–9)^ — for example, retailers’ knowledge and skills, retailer-consumer relationship, consumer demand and food preferences, and store infrastructure including product availability, space, and resources among others.

The effectiveness of similar food retail interventions to promote the purchase of healthy foods, as well as factors that influence the uptake and success of such interventions in more complex food retail settings, is unclear. Food retail environments where multiple food retail outlets are operating in close proximity including in shopping malls, on high streets, in airport food lounges, or within institutions such as a hospital or an educational institution are more complex than a single food retail outlet.^(10,11)^ This is because such settings typically include a governing body that not only leases out the space to food retail outlets but may also guide and monitor the activities of the food retail outlet in a desired direction. Further, in settings with multiple food retail outlets, there is likely to be a competitive nature between the food outlets (inner settings) which can be affected by external structures (outer settings) and processes and can impact the retailer’s capacity to embrace business risk and become an early adopter of healthy food options.^(12)^ These interactions between the inner and outer settings create complexity, which is likely to vary in different settings. For example, a mall compared with a hospital will typically have different institutional practices and policies, organisational commitment to healthy food retail, culture, and customer demands.^(13)^ There is some evidence suggesting that implementing multi-component population-based interventions such as education, policy, or advocacy at multiple levels of the socio-ecological model (individual, societal, and environmental) may improve health outcomes.^(14)^ However, the evidence is inconclusive on the effectiveness of this type of intervention when implemented in settings with multiple food retail outlets.

As the implementation success of any intervention largely depends on the context in which it is implemented, and because most food retail outlets do not exist in isolation, it is imperative to understand the factors that influence the implementation and effectiveness of interventions in complex food retail settings. This systematic review aimed to (1) identify and describe complex food retail settings where interventions were implemented to promote the healthiness of foods purchased, (2) synthesise the evidence on the effectiveness of the interventions implemented in complex food retail settings, and (3) identify enablers and barriers associated with the implementation of the interventions in complex food retail settings. Evidence from this review will offer an understanding of how and what healthy food retail interventions are implemented in more complex settings. This will then help improve the design, uptake, and compliance of the healthy food retail interventions by multiple food retail outlets situated in a particular setting, for a positive impact on population diet and health.

## Methods

A review protocol was developed *a priori* and registered in PROSPERO (The International Prospective Register of Systematic Reviews; registration number CRD42021258235).^(15)^ For reporting of this systematic review of reviews, the PRISMA (Preferred Reporting Items for Systematic Reviews and Meta-Analyses) guideline^(16)^ was followed.

### Search strategy

A complex food retail setting is defined as a commercial or non-commercial establishment whose primary purpose may or may not be to sell food and drinks (e.g. hospitals/health services, sports, and recreational centres; shopping strip), and that includes multiple food retail outlets operating within the setting. A range of keywords and MeSH terms (Appendix A) were chosen to capture multiple food retail outlets across diverse settings, globally to capture complex food retail settings. Public health experts in the United States (US), United Kingdom (UK), and Australia were consulted to identify terminologies used to refer to complex food retail settings in different countries. For example, ‘shopping strips’ in Australia are often referred to as ‘high streets’ in the UK and ‘street intersections’ in the US. The search was conducted in four databases: Medline Complete, Global Health, Embase and Business Source Complete. Database search terms were categorised under the following hedge terms: settings with multiple food retail outlets, healthy food retail interventions, and healthy eating measures. Using the same search terms, an online search using Google Advanced was also conducted to identify grey literature published from inception up to December 2022 to expand the scope of the search. The search was limited to the first 100 uniform resource locators (URLs) depending on relevancy. A citation search of included papers was performed (‘forward search’), and the reference lists of all relevant articles were searched to capture any potentially relevant paper missed by electronic search (‘backwards search’). All articles (English language only) identified were subjected to selection criteria as described in the below section.

### Study selection

A modified PICOS (population, intervention/exposure, comparison, outcome, and study context) criterion was developed to inform the study selection (Table 1). Articles were eligible for inclusion if they examined interventions (including but not limited to the ‘4Ps’ of the marketing mix strategies, i.e. price, promotion, product, and placement)^(3)^ implemented across multiple food retail outlets within a setting to promote the healthiness of foods purchased. Information on enablers and barriers to implementation and effectiveness of food retail interventions were either extracted wherever reported or inferred from the discussion section of the selected papers.

**Table 1.** PICOS criteria

Criteria	Inclusion	Exclusion
Population	Studies that included and identified multiple food retail outlets within a setting *(defined as an establishment primarily engaged in retailing a general line of food, e.g. cafeteria, grocery store located in a hospital, university, etc.)*.	Single food retail outlets operating or not operating within a setting, for example, supermarkets, dining halls or food pantries, online food retail outlets, and online experiments. No exclusions were based on race, culture, ethnicity, or geographical location of the food retail or retailers.
Intervention/exposure	In-store food retail interventions (including but not limited to 4Ps, i.e. price, promotion, product, and placement) to promote healthy food purchased within food environment settings with food retail outlets.	Studies that do not include a relevant food retail intervention or online experiments.
Comparator	No restrictions	No restrictions
Outcome	Primary outcome: Studies reporting on the effectiveness of food retail interventions on healthy food purchased measured as food purchased, food consumed, and dietary intake. Secondary outcome: Studies reporting on implementation outcomes (e.g. adoption and engagement)	Studies that only reported on changes in knowledge and attitude; intention to purchase or consume healthy food.
Type of studies	All studies published in the English language from inception to December 2022	Commentaries, conference abstracts, editorials, laboratory-based or modelling studies

After duplicate articles were removed, titles, abstracts, and full texts were examined against the inclusion and exclusion criteria by two authors independently. Any discrepancies were resolved via consensus between the two screening authors.

### Data extraction

Data was extracted from all included articles by two authors independently, and their results were compared. Data were collated into a predetermined matrix table that included publication details (author, title, year, study design, and aim), type of setting, type and number of food retail outlets, type of food sold, mention of any governing policy, and type and description of intervention including behaviour change theories underpinning the intervention, information on formative research conducted to inform the intervention, and key findings on the effectiveness of healthy food retail interventions on the healthiness of foods purchased (measured as food purchased, food consumed, or dietary intake), evaluation outcomes, and reported enablers and barriers to implementation healthy food retail interventions.

### Quality appraisal

To ensure credibility, relevance, and value, each included article was critically appraised independently by two authors using the Effective Public Health Practice Project (EPHPP) Quality Assessment Tool.^(17)^ The rating was based on the quality assessment across all six domains of the EPHPP tool — selection bias, study design, confounding, blinding, data collection, and withdrawal/dropouts.

### Data analysis

A narrative synthesis describing the characteristics of complex food retail settings that implemented interventions to promote the healthiness of foods purchased, the effectiveness of interventions implemented, and the associated enablers and barriers to implementation across different types of settings with multiple food retail outlets was conducted.

## Results

**Fig. 1.**
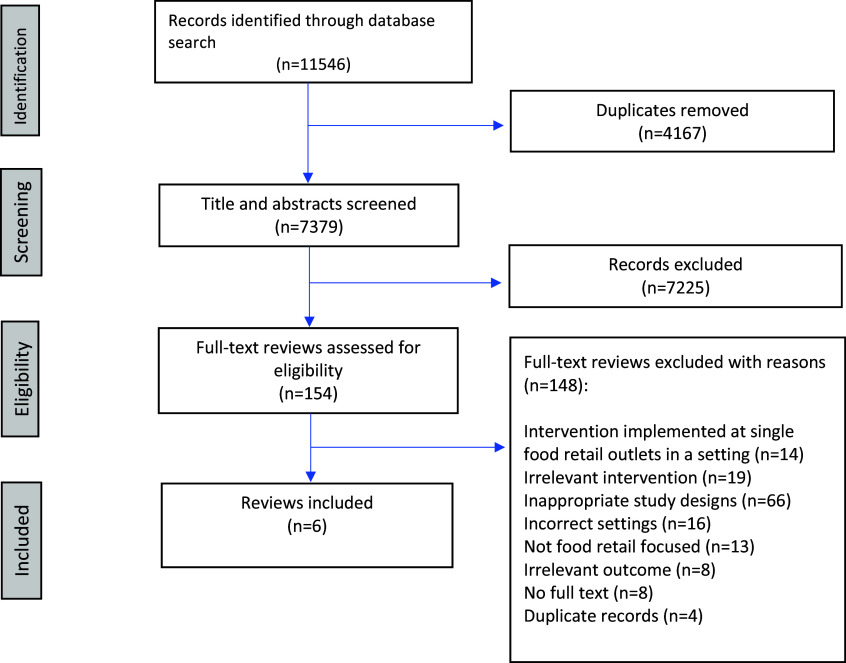
PRISMA flowchart.

A total of 11,546 published peer-reviewed articles were identified from database searches. Following the removal of duplicates (n=4,167), title and abstract screening was conducted for 7,379 articles. Of these, 154 articles underwent full-text review. Following full-text screening, 148 articles were excluded based on reasons listed in the PRISMA flowchart (Fig. 1: PRISMA flowchart). A total of six distinct articles that implemented food retail interventions in two different settings to promote the healthiness of foods purchased were considered eligible for inclusion in the review. No grey literature met the eligibility criteria and hence was not included.

### Study design and study participants

Two of the six included studies were quasi-experimental studies^(18,19)^ (with no randomisation and no control group), of which one was conducted in two canteens in a university in Belgium^(18)^ and the other at two cafeterias in a hospital in the US.^(19)^ One of the six was a quasi-experimental study (with no randomisation) with a control group conducted at two cafeterias in a hospital in Canada,^(20)^ one a cluster randomised controlled trial at 30 shops (food outlets) in a hospital in the UK,^(21)^ one a non-experimental programme evaluation at three food outlets in a university in the US,^(22)^ and one a cross-sectional observational study conducted at five food outlets in a university in New Zealand (NZ).^(23)^

Within studies conducted in universities, participants were largely students and staff, aged 17–35 years, and the sample size ranged from 111 to 244 participants. Participants in studies conducted in hospitals included staff and visitors, aged 18 years and above (only adults participated in the studies), and the sample size ranged from 1013 to 2800 participants. Table 2 provides a detailed description of the characteristics of the six included studies.

**Table 2.** Characteristics of the six included studies grouped by settings

Publication details	Type and number (n) of food retail outlets	Type of food sold at the food retail outlets	Existing governing policy (yes/no)	Type of intervention	Key findings		Risk of bias
					Effectiveness of food retail interventions on diet	Enablers and barriers to implementation^a^	
University settings							
Hoefkens et al. 2011^(18)^ Belgium Quasi-experimental studies (with no randomisation and no control group)	Canteens (n=2)	Four protein sources (e.g. meat), one or two warm sauces, two cooked vegetables, one salad, and five carbohydrate components (e.g. French fries); dressings, fruit, other desserts, and drinks	Yes (Food preparation methods and menus were standardised in all canteens of the universities)	At the point of purchase, stars based on energy content, saturated fat, sodium, and vegetable portions are posted on large poster boards at the entrance of the canteens and next to example dishes at the buffet counter. Besides the number of stars, noncomplying nutrients or food groups were posted in a red font and followed by an exclamation mark or verbal descriptor. PROMOTION	The proportion of meals chosen in the different star-rating categories remained constant after posting the nutrition information (P=0.820). Posting nutrition information did not affect meal choices and nutrient intakes.	*Enabler:* Commitment to promote health; organisational policy	Weak quality
Magdaleno et al. 2021^(22)^ United States Non-experimental programme evaluation (process and impact evaluation)	Markets (n=3)	A variety of food and beverage options including many ‘grab and go’ items in cold cases.	No	Front-of-pack labelling: The sticker is bright red with a heart graphic and a slogan that states ‘Live Well [the university’s mascot]’. The items that were selected to display the sticker contained one or more of the following nutritional benefits based on FDA guidelines: 100% whole grain, lean protein, fruits, vegetables, high fibre, low sodium, low saturated fat, and/or low sugar. PROMOTION	Participants did not purchase an item with the sticker.	*Enabler:* Collaborations and leadership at the organisation level	Weak quality
Roy et al. 2021^(23)^ New Zealand A cross-sectional observational study	Food outlets (n=5)	Meals (food and beverages)	No	Price-reduced meals: The ‘Budgie Meal’ is a price-reduced meal option available at most food outlets, offering staff and students the opportunity to purchase a substantial meal from an array of cuisines across campus. The meals are $6⋅50 or under and consist of protein, vegetables, and carbohydrates. PROMOTION, PRICE	The ‘Budgie Meal’ had higher sales volumes at each outlet than other items.	*Enabler:* Commitment to promote health *Barrier:* No organisational support and governance; Retailer’s profit-oriented approach and unwillingness to host interventions	Weak quality
Hospital settings							
Vanderlee et al. 2014^(20)^ Canada Quasi-experimental studies (with no randomisation but a control group)	Cafeteria (n=2)	Overall nutrition intake	Yes (Hospital check criteria)	• Five digital menu boards with prominent displays of nutrition information at the point of sale next to the price of items on the menu board, featuring information on energy (calories), sodium, saturated fat, and total fat • A health logo (an apple with a check mark) for items that met the developed nutritional standards; • A Healthier Menu Plus Sante at the entrance to the cafeteria highlighting healthier menu items available on the menus; • An educational campaign (posters and pamphlets) promoting the programme • Reformulated recipes for some food items and removed the deep fryer from the cafeteria PROMOTION	Nutrition information on menus and improved nutrition profile of food offerings were positively associated with substantial reductions in energy, sodium, and fat consumption.	*Enabler:* Commitment to promote health; organisational policy *Barrier:* Retailer’s profit-oriented approach	Weak quality
Patsch et al. 2016^(19)^ United States Quasi-experimental studies (with no randomisation and no control group)	Cafeteria (n=2)	Burgers and salads	Yes (‘Healthy food’ punch card policy—where employees earned a free healthy meal after 10 healthy purchases)	Marketing and price incentives/disincentives for healthy and unhealthy items, with a 35% price differential. Point-of-purchase marketing included the following: (1) Better Bites (BB) logo on all food items meeting nutritional criteria and (2) signage highlighting taste, cost, and health benefits. Intervention strategy was developed by combining evidence-based practices with hospital-specific formative research, including key informant interviews, the Nutrition Environment Measures Study in Restaurants, hospital employee surveys, and nutrition services staff surveys. PROMOTION, PRICE	Average weekly turkey burger sales increased 13-fold (10.85–145.59); healthy salads were popular at baseline and intervention. Cafeteria gross sales and burger profit (P < .001) increased at both cafeterias.	*Enabler:* Commitment and moral obligation to promote health; leadership at the organisation level; organisational policy	Weak quality
Allan et al. 2020^(21)^ United Kingdom Cluster randomised controlled trial	Shops (n=30)	Snack food	Yes (Healthcare Retail Standards)	Theory-based point of purchase prompts (a form of cognitive nudge) — sign displaying all the available single-serve snacks in order from lowest calorie on the left to highest calorie on the right. Intervention design was underpinned by different components of behaviour change technique^(24)^ to cognitively simplify healthier snack choices by facilitating cross-product comparison. Intervention strategy was developed by formative research including consultation with a multidisciplinary team of psychologists, public health scientists, and nutritionists (formative research) and a successful pilot study. PROMOTION	Snacks purchased from intervention sites were on average significantly lower in calorie and sugar.	*Enabler:* Commitment and moral obligation to promote health; collaborations and leadership at the organisation level; organisational policy *Barrier:* Retailer’s profit-oriented approach; instore competition between health versus profit-oriented interventions	Weak quality

a Inferred from the discussion section of the included studies.

### Characteristics of complex food retail settings

No study referred to the settings in which the studies were conducted as being a multiple food retail outlet setting or similar. Broadly, the studies described the characteristics of the university and hospital settings regarding the size, location, population demographics, characteristics of food retail outlets, and pre-existing food policy/health guidelines. Two studies^(22,23)^ reported that the universities were in urban areas and considered themselves as a large public higher education setting hosting thousands of students and staff with middle-to-high socioeconomic levels. Regarding the characteristics of multiple food retail outlets where the interventions were tested, the number of food outlets in the university and hospital settings varied from two to thirty. The food outlets varied in terms of size, catering for <100 to >1000 consumers, types of food sold (from pre-packaged foods, hot meals, salads, snacks, desserts, hot sides, and fruits), vendor properties (trading hours generally ranged from morning to evening on weekdays, except for food retail outlets at one hospital^(19)^ that remained open on weekends and served food up to midnight), and customers (staff, students, and visitors). In two studies,^(19,20)^ the hospitals where the interventions were implemented were large hospitals located in urban or metropolitan city areas. All studies conducted in hospitals (n=3) and one in a university mentioned an existing policy in place (including healthcare retail standards,^(21)^ hospital check criteria,^(20)^ ‘healthy food’ punch card policy,^(19)^ and preparing standardised meals^(18)^) to influence the type of food sold at food retail outlets within their settings. Only two studies were conducted in hospitals in the UK, and the US reported that their intervention strategies were developed through some form of formative research including survey data,^(19)^ qualitative interviews,^(19,21)^ and a pilot study.^(21)^ No study explicitly reported on whether their intervention was underpinned by a health behaviour change framework. However, the authors of one study conducted in a hospital in the UK^(21)^ stated that their intervention design included different behaviour change techniques^(24)^ to cognitively simplify healthier snack choices by facilitating cross-product comparison.

### Effectiveness of healthy food retail interventions to promote the purchase of healthy foods, implemented in complex food retail settings

All studies tested the effectiveness of marketing mix strategies,^(3)^ either as a standalone strategy or in combination with other strategies on healthy food purchased. The healthiness of the food purchased was collected using objective sales data from the participating food retail outlets. Three studies measured the energy (calorie or kilojoule) content of purchases,^(18,20,21)^ one measured the average and proportional change in sales,^(19)^ and two studies measured food purchased through a customer intercept survey.^(22,23)^

Two of the four^(18,20–22)^ studies that tested the impact of a promotion strategy (defined as food and beverage marketing practices that promote products that adhere to healthy dietary guidelines) reported conflicting results. Promotion interventions delivered at food outlets in universities in the US^(22)^ (as health star ratings) and in Belgium^(18)^ (as an infographic and slogan front-of-package labelling) reported no change in the proportion of healthy meals purchased. In contrast, promotion interventions delivered at food outlets in hospitals in Canada^(20)^ and the UK^(21)^ reported healthier food purchases. In Canada,^(20)^ digital menu boards with prominent displays of nutrition information coupled with an educational campaign (posters and pamphlets) to encourage customers to make healthy decisions at the point of choice or point of purchase were associated with a substantial reduction in foods purchased with high energy (-21%; P<0.001), high sodium (-23%; P<0.001), high saturated fat (-33%; P<0.001) and total fat (-37%; P<0.001) content. In the UK, an intervention trial in thirty hospitals (fifteen intervention and fifteen control) tested a warning label sign displaying all of the available single-serve snacks (>1 million) in order from lowest calorie on the left to highest calorie on the right resulted in significant reductions in snacks purchased with high energy content (total energy and sugar).^(21)^ The authors stated that the intervention was designed to cognitively simplify healthier snack choices by facilitating cross-product comparison.

Two studies that tested a combination of promotion and price manipulation strategies at two cafeterias in a hospital in the US^(19)^ and five food outlets in a university in NZ^(23)^ resulted in significant reductions in unhealthy food purchases. In the two hospital cafeterias, the promotion component included a ‘Better Bites’ logo on food items including burgers (traditional hamburger vs ‘Better Bites’ turkey burger), salads (traditional salad vs ‘Better Bites’ healthy salad)), and signage highlighting taste, cost, and health benefits. The prices of healthy items were decreased to incentivize purchases, and prices of unhealthy items were increased to disincentivize purchases at the point of purchase. A 35% intervention price differential was applied. The intervention resulted in a 13-fold increase in the average weekly Better Bites turkey burger sales and increased popularity of the Better Bites salads, leading to increased gross sales and profit at both cafeterias.^(19)^ A study evaluating five food outlets at a university in NZ found that the promotion and price-reduced meal choice initiative ‘Budgie Meal’ resulted in higher purchases of these meals.^(23)^

### Associated enablers and barriers to implementation of food retail interventions in complex food retail settings

None of the six included articles reported on the enablers and barriers to the implementation of the various food retail interventions. One study conducted in a university in the US^(22)^ included process evaluation outcome assessment on the completeness of programme delivery and the degree to which participants were engaged with the intervention via audits and surveys with customers. Some authors speculated on enablers and barriers in the discussion section of their article to explain the outcomes of the study.

### Quality appraisal

All six studies included two or more weak quality ratings across the tool criteria and therefore were evaluated as weak in the global rating. Briefly, five studies were rated as moderate or weak quality due to high selection bias^(18,19,21–23)^ and weak study design.^(18–20,22,23)^ Three studies did not adjust for, or did not report on, confounders and were rated as weak. Three other studies^(19–21)^ were rated as strong for treating confounders. Blinding in all studies was assessed as weak, except for one study that was rated as moderate.^(21)^ Data collection was primarily through either sales data (n=4) or self-reported data (n=2). The validity and reliability of the data collection tool were reported in only one study.^(22)^ Only three studies reported achieving more than 60% of participants completing the study.^(18,20,22)^ For details on the assessment across each of the tool criteria, please refer to Appendix B.

## Discussion

This is the first systematic review to summarise the evidence on food retail interventions to promote the purchase of healthy foods implemented in complex food retail settings, that is, where multiple food retail outlets operate together in close proximity in a particular setting. We identified six studies where a relevant food retail intervention had been implemented in a complex food retail setting; however, none of the studies identified the surrounding food retail outlets in their setting as relevant to the implementation or effectiveness of the interventions. The intervention outcomes along with associated enablers and barriers were neither analysed nor discussed in the context of the interrelationship between the setting and the multiple food retail outlets.

The six included studies were conducted in university and hospital settings where two marketing mix strategies, promotion (as a standalone strategy) and price (in combination with promotion), were implemented in multiple food retail outlets and their effect on food purchased was assessed. University and hospital settings were defined here as complex food retail settings as they included multiple food outlets such as cafés, restaurants, and vending machines, offering diverse food options to their consumers. The studies described these settings regarding their size, location, population demographics, and characteristics of food retail outlets more broadly. Furthermore, there was limited description of formative research and process evaluation, to inform intervention design and development. While previous literature suggests that these characteristics are likely to influence the implementation of interventions in single food retail outlets,^(12,25)^ there is no comparable literature to confirm whether these are also characteristics relevant to complex food retail settings. This is critical information for policymakers and researchers to better plan the implementation of the intervention specific to such settings to achieve maximum success.^(26)^

Studies^(20,21)^ that implemented promotion interventions in the form of warning labels and displayed nutrition information including a health logo on a digital menu board at food outlets in hospitals reported a positive effect on healthy food purchasing behaviour. Studies^(19,23)^ that implemented a combination of promotion and price (in the form of a display sign highlighting taste, cost, and health benefits) at food outlets in both university and hospital settings reported a positive effect on healthy food purchasing behaviour and overall sales of healthy foods. These findings are consistent with the findings of previous studies^(4,27–30)^ that have reported positive effects of combined promotion and price strategies on the healthiness of foods purchased implemented in small (i.e. convenience stores) and large (i.e. supermarkets or recreation and sports facilities) settings but with single food retail outlets. While some of the studies in our review reported the presence of an existing health-enabling food policy,^(18–21)^ none mentioned their impact on the implementation of the interventions. Furthermore, the studies did not report on whether and how the intervention design and implementation factors were adapted according to the characteristics of complex food retail settings. This is important as the settings may have their own culture, structures, policies, and practices, serving a certain interest,^(12,31)^ and this may impact the level of uptake of the interventions or intervention success in multiple food retail outlets. For example, there was no discussion of the impact of a potential competitive disadvantage if an outlet becomes an early adopter in their broader setting.

While no study in our review analysed enablers and barriers to the implementation of food retail interventions in the context of complex food retail settings, some authors made inferences in their discussion sections on potential enablers and barriers that influenced intervention outcomes. Factors such as collaboration and leadership^(19,21,22)^ at the setting level and existing health-enabling food policy^(18–21)^ were assumed as enablers that led to the positive impact of interventions on the outcomes. Barriers to implementation were speculated to be the lack of organisational support to implement healthy food policy^(23)^ at the setting level and the retailer’s concern for profitability.^(19–21,23)^ Several reviews have previously summarised similar factors (from retailer and consumer perspectives) influencing the implementation of food retail interventions at a single food retail outlet.^(6,12)^ However, factors influencing the implementation of food retail interventions specific to complex food retail settings remain an area open to exploration. To advance knowledge in this context, using a combination of the Consolidated Framework for Implementation Research (CFIR) tool^(32)^ and socio-ecological model^(6)^ can be useful to not only identify the characteristics of outer setting (i.e. the external influence of the organisation on implementation of the intervention) and inner setting (i.e. characteristics of the food retail outlets implementing interventions) domains but also explain the interrelationship between the factors within the two domains. Moreover, CFIR comprehensively encompasses both barriers and enablers to the implementation of interventions, contributing to an in-depth understanding of the processes involved. For example, in Singapore, strong leadership in the Health Promotion Board’s Healthier Dining Programme initiative in 2019^(33,34)^ (outer setting) led the food retailers within some hawker centres (i.e. a complex food retail setting) to cook with healthier amounts of oil and salt and shifting the culture of the hawker centres (inner settings) towards more health-promoting. Given the limited description of formative research to inform the implementation of the intervention in the included studies, future studies could better report on steps and processes undertaken with key stakeholders to unpack the complex interplay between the settings and food outlet level to help inform the implementation strategies for effective and sustained implementation of interventions in complex food retail settings.

The overall lack of evidence on complex food retail settings that implemented interventions to promote the purchase of healthy foods illustrates the need for better investigation of the characteristics of complex food retail settings (beyond universities and hospitals) for context and factors (at the setting level and the retailer level) influencing the procurement, preparation, and provision of food at multiple food retail outlets for three reasons. First, to enable identifying and undertaking a shared/collaborative approach (between key actors — organisational contractors and retailers) to developing a comprehensive plan for programme and policies tailored (to the context) to promote healthy food choices culture within their settings.^(31,35)^ Second, understanding the context and factors (at the setting level and the retailer level) influencing the implementation of interventions in complex food retail settings is important to identify appropriate ways to mitigate context-specific challenges and harness opportunities to facilitate sustained implementation of healthy food retail interventions.^(11,36)^ Last, better characterising complex food retail settings can help authorities in power such as local governments to develop standardised healthy food policies that can be implemented consistently across the multiple food retail outlets operating within a setting to make a population-level impact.^(31)^ For example, healthy food policies can be linked to lease agreements, contracts, and tenders applicable to the food outlets within a setting.^(37)^ Existing resources such as the Victorian ‘Healthy Choices: healthy eating policy and catering guide for organisations’^(38)^ can be useful resources to adapt and implement as a healthy food policy in a complex food retail setting. Another likely benefit of implementing a setting-level food policy is that consumers are less likely to indulge in compensatory unhealthy food purchases^(39)^ in the vicinity of complex food retail settings. However, this warrants future investigation.

### Strengths and limitations

A strength of this review is the identification and inclusion of a broad range of search terms to capture complex food retail settings. The main limitation is that only six studies of weak quality were identified and included that lacked randomisation, lacked assessor and participant blinding raising concerns regarding internal validity, and used a convenience sample of students and hospital visitors which may not be representative of the general population. However, within a real-life university or hospital setting, quasi-experimental studies are deemed more appropriate considering problems of contamination. Second, the review only found universities and hospitals as two examples of complex food retail settings, the findings of which may not be generalisable to other complex food retail settings such as shopping strips. Last, we excluded non-English language papers and studies where the authors did not report the settings in which the interventions were implemented; this may have led to the exclusion of studies with insights relevant to complex food retail settings.

### Research implications

First and foremost, good quality studies investigating the complex food retail setting, and giving consideration to the nature of that environment, are needed. Exploring complex food retail settings regarding their governance structure, existing nutrition policies, and the interaction between factors at the settings and the retailer level is needed to understand the implementation of healthy food retail interventions in complex food retail settings. This will also enable the development of context-specific support for complex food retail settings. Food retail interventions need to be tested for their fit and evaluated (using process and outcome evaluation measures) to better understand the barriers and enablers to implement within the context of complex food retail settings. Identifying ways to mitigate barriers to successful implementation of interventions will then be the logical next step. Multi-stakeholder collaboration between public health researchers, policymakers, and the various actors within complex food retail settings, including consumers, is needed to collectively inform public health interventions.^(40)^ Future research can investigate existing food environment monitoring and benchmarking initiatives^(36)^ in the context of complex food retail settings. This may also identify potential levers of success to create healthy complex food retail settings. Finally, with an increasing number of food retail outlets that co-exist both in conventional brick-and-mortar-type settings and within emerging online food retail settings,^(41)^ there is an opportunity to adapt the learnings from complex food retail settings to influence the emerging online forms of food environment towards health promotion in their infancy.

## Conclusion

This systematic review demonstrates a significant gap in the existing evidence regarding the clear conceptualisation and description of healthy food retail interventions implemented in settings where multiple food retail outlets are co-located (i.e. complex food retail settings). Universities and hospitals are two examples of such settings identified in this review where healthy food retail interventions have been implemented. Evidence suggests that healthy food retail interventions including promotion and price strategies can lead to healthier purchasing behaviour among consumers in university and hospital settings where multiple food retail outlets are co-located. However, exploring complex food retail settings regarding their governance structure, existing nutrition policies and other characteristics are fundamental to inform the development and successful implementation of healthy food retail interventions at the settings level.

## Supporting information

Gupta et al. supplementary material 1 Gupta et al. supplementary material

Gupta et al. supplementary material 2 Gupta et al. supplementary material

## Supplementary material

The supplementary material for this article can be found at https://doi.org/10.1017/jns.2024.52.
