# Supplementary material for: A systematic review of the implementation of healthy food retail interventions in settings with multiple food retail outlets (complex food retail settings)
Source: J Nutr Sci. 2024 Sep 18;13:e31. doi: 10.1017/jns.2024.52 (PMC11418081; doi:10.1017/jns.2024.52)
Supplement: Gupta et al. supplementary material 1 — Gupta et al. supplementary material [file S2048679024000521sup001.docx]

APPENDIX A

| Limits | Food retail outlet (type/ setting) TI | Intervention foci (TIAB) | Outcome (TI) |
| --- | --- | --- | --- |
| English  Only  Inception to Dec 2022 | TI “Food industry” OR AB “Food industry” OR MH “Food industry” OR TI “Airport lounge*” OR AB “Airport lounge*” OR MH “Airport lounge*” OR TI Universit* OR AB Universit* OR MH Universit* OR TI campus OR AB campus OR MH campus OR TI College* OR AB College* OR MH College* OR TI institution* OR AB institution* OR MH institution* OR TI “Industrial Organisation*” OR AB “Industrial Organisation*” OR MH “Industrial Organisation*” OR TI “Food court*” OR AB “Food court*” OR MH “Food court*” OR TI “Shopping strip*” OR AB “Shopping strip*” OR MH “Shopping strip*” TI Mall* OR AB Mall* OR MH Mall* OR TI Market* OR AB Market* OR MH Market* OR TI hospital* OR AB hospital* OR MH hospital* OR TI “street intersection*” OR AB “street intersection*” OR MH “street intersection*” OR TI “farmers market*” OR AB “farmers market*” OR MH “farmers market*” OR TI “high street*” OR AB “high street*” OR MH “high street*” OR TI ((“Food retail*” OR “food outlet*” OR “take away” OR takeaway OR restaurant* OR café* OR vend* OR takeout) N3 (Universit* OR campus OR College* OR institution* OR “Industrial Organisation*” OR Mall* OR Market* OR hospital* OR “high street” OR “shopping strip”)) | product OR place* OR profile OR portion* OR price* OR pricing OR OR promot* OR priming OR prompt* OR proximity OR availab* OR discount* OR voucher* OR incentive* OR bonus* OR reward* OR coupon* OR token* OR rebate* OR refund* OR access* OR display* OR layout OR strateg* OR advert* OR activit* OR initiative* OR program* OR "food quality" OR reformula* OR modif* OR adapt* OR recipe* OR “Point of purchase” OR “Point of sale” OR intervention* | Food OR nutrition OR diet OR healthy* OR consum* OR purchas* OR intake OR eating OR sustainab* OR (energy or calorie*) N3 (intake* OR chang* OR pattern*) |
