# Supplementary material for: A systematic review of the implementation of healthy food retail interventions in settings with multiple food retail outlets (complex food retail settings)
Source: J Nutr Sci. 2024 Sep 18;13:e31. doi: 10.1017/jns.2024.52 (PMC11418081; doi:10.1017/jns.2024.52)
Supplement: Gupta et al. supplementary material 2 — Gupta et al. supplementary material [file S2048679024000521sup002.docx]

| Author(s), Year | Selection Bias  (Overall) | Study design  (Overall) | Confounders  (Overall) | Blinding  (Overall) | Data collection  (Overall) | Withdrawal and dropouts  (Overall) | Global rating  (Overall)* |
| --- | --- | --- | --- | --- | --- | --- | --- |
| UNIVERSITY SETTING |  |  |  |  |  |  |  |
| Hoefkens et al. 2011^1^ | Moderate | Moderate | Weak | Weak | Moderate | Moderate | Weak |
| Magdaleno et al. 2021^2^ | Weak | Weak | Weak | Weak | Strong | Strong | Weak |
| Roy et al. 2021^3^ | Weak | Weak | Weak | Weak | Weak | Weak | Weak |
| HOSPITAL SETTING |  |  |  |  |  |  |  |
| Allan et al. 2020^4^ | Moderate | Strong | Strong | Moderate | Weak | Weak | Weak |
| Patsch et al. 2016^5^ | Moderate | Moderate | Strong | Weak | Weak | Weak | Weak |
| Vanderlee et al. 2014^6^ | Strong | Moderate | Strong | Weak | Weak | Strong | Weak |

APPENDIX B: Study quality assessments using EPHPP tool

* Strong (no weak ratings), Moderate (one weak rating), Weak (two or more weak ratings)

REFERENCES

1. Hoefkens, C, Lachat, C, et al. Posting point-of-purchase nutrition information in university canteens does not influence meal choice and nutrient intake. *The American journal of clinical nutrition*, 2011, 94, 562-570.

2. Magdaleno, L, Rolling, T, et al. Evaluation of a front-of-pack food labeling intervention on a college campus. *Journal of American college health : J of ACH*, 2021, DOI: 10.1080/07448481.2021.1970563, 1-9.

3. Roy, R and Harrington, K. Effectiveness of price-reduced meals on purchases among university young adults. *Journal of nutritional science*, 2021, 10, e94.

4. Allan, JL and Powell, DJ. Prompting consumers to make healthier food choices in hospitals: a cluster randomised controlled trial. *The international journal of behavioral nutrition and physical activity*, 2020, 17, 86.

5. Patsch, AJ, Smith, JH, et al. Improving Healthy Eating and the Bottom Line: Impact of a Price Incentive Program in 2 Hospital Cafeterias. *American journal of health promotion : AJHP*, 2016, 30, 425-432.

6. Vanderlee, L and Hammond, D. Does nutrition information on menus impact food choice? Comparisons across two hospital cafeterias. *Public health nutrition*, 2014, 17, 1393-1402.
